# Are Hong Kong and Taiwan stepping‐stones for invasive species to the mainland of China?

**DOI:** 10.1002/ece3.3818

**Published:** 2018-01-15

**Authors:** Jianbo Lu, Shao‐peng Li, Yujia Wu, Lin Jiang

**Affiliations:** ^1^ Qianjiang College Hangzhou Normal University Hangzhou China; ^2^ School of Biological Sciences Georgia Institute of Technology Atlanta GA USA; ^3^ College of Life and Environmental Sciences Hangzhou Normal University Hangzhou China

**Keywords:** biogeographical approach, biological invasions, China, colonial history, island, transport network

## Abstract

Understanding the origins and introduction pathways of invasive species is a fundamental issue for invasion biology, which is necessary for predicting and preventing future invasion. Once an invasive species is established in a new location, this location could serve as a stepping‐stone for further invasions. However, such “stepping‐stone” effect has not been widely investigated. Using the published literature and records, we compiled the first found locations of 127 top invasive species in China. Our study showed that the most common landing spots of these invasive species were Hong Kong (22 species) and Taiwan (20 species), which accounted for one‐third of the invasive species in China. Our analysis revealed that the invasive species in mainland China were more likely to transport from Hong Kong than Macau, a neighboring region with a similar area and colonial history. Similarly, more invasive species were also first landed on Taiwan than Hainan, a nearby island sharing similar climate conditions. Together, our findings indicate that Hong Kong and Taiwan are the most important stepping‐stones for invasive species to the mainland of China and suggesting that the increasing trade exchange of China's coastal ports constitutes a potential risk for the spread of more invasive species. We suppose that they would be the future stepping‐stones for invasive species to the mainland of China and these coastal ports regions where improved biosecurity is needed now.

## 1 INTRODUCTION

It has been almost 60 years since Charles Elton published his classic book on biological invasions, *The Ecology of Invasions by Animals and Plants* (Elton, 1958; Ricciardi & Maclsaac, 2008; Richardson, 2011; Richardson & Pysek, 2008). Ever since then biological invasions have become serious global environmental problems as a result of the globalization (Perrings, Dehnen‐Schmutz, Touza, & Williamson, 2005). Nowadays, understanding the geographical distributions and spread pathways of invasive species has become a central goal of invasion biology and could benefit the prediction and prevention of future invasions (Cassey, Vall‐Llosera Camps, Dyer, & Blackburn, 2015). For example, more than half of the naturalized plant species and invasive species originate from North and South America (Jiang et al., 2011; Xu et al., 2012). A comprehensive assessment of naturalized vascular plants worldwide has already been performed recently, which revealed that Northern Hemisphere is the major donors of alien species to other continents (van Kleunen et al., 2015). It has been suggested that anthropogenic effects, especially global trades and transports, were major drivers of spread of invasive species worldwide. For example (Banks, Paini, Bayliss, & Hodda, 2015; Seebens et al., 2015), it was reported that the pet trade was a driver of introduction and establishment in alien birds in Taiwan (Su, Cassey, & Blackburn, 2016). It was also pointed that colonization pressure is key to understanding alien bird species richness at the global scale, alien bird species richness is currently highest at midlatitudes and is strongly determined by anthropogenic effects (Dyer et al., 2017). Global trade and transport network topology were used to study the geographical spread of invasive species and enable the development of more effective strategies to prevent invasions, utilizing this new insight and tools in a systemic approach can help decision‐makers in managing threats to national and regional biosecurity and in safeguarding the world's natural and managed ecosystems (Banks et al., 2015). However, our knowledge of the spread pathways and landing spots of invasive species is still very limited, which may hinder our ability to make up effective policy and management strategies for invasion prevention (Dawson et al., 2017; Russell & Blackburn, 2017).

Once an invasive species successfully established in a new location via long‐distance dispersal, such invaded location could serve as a stepping‐stone for further invasions to the nearby locations. Such “stepping‐stone” effect has been observed for the spread and dispersal of invasive species in aquatic and terrestrial ecosystems (Buchan & Padilla, 1999; Suarez, Holway, & Case, 2001). With the growth in international trade and travel activities, some invasion hotspots would act as stepping‐stones to determine the rate and spatial pattern of spread of invasive species (Floerl, Inglis, Dey, & Smith, 2009). However, unlike the extensive research on the geographical origin of invasive populations, few studies have focused on the first landing places of alien species in a new area and their introduction pathways, making it difficult to test the “stepping‐stone” hypothesis.

As the third largest country and the biggest trading nation in the world, China faces grave challenges of biological invasion. The economic losses caused by invasive alien species in China are estimated to be more than 14 billion USD per year (Xu et al., 2006). So far, a number of studies have investigated the origins, geographical distribution, invasion mechanism, ecological effects, and detrimental economic impacts of biological invasions in China (Liu, Liang, Liu, Wang, & Dong, 2005; Liu et al., 2006; Weber, Sun, & Li, 2009; Xu et al., 2006, 2012). Further, the province‐based distribution of invasive plants in China has also been published (Feng & Zhu, 2010; Liu et al., 2005), making it possible to identify locations where invaders first establish their populations. However, studies on the first landing places of invasive species in China and their spread pathway with a biogeographical approach are still lacking.

Using the published literature and records, we compiled the first landing places of 127 top invasive species in China, which provide an important dataset for revealing the potential stepping‐stones for these invaders. We further compared the landing patterns of two trade hubs (Hong Kong and Taiwan) with their neighboring regions with similar area and climate conditions (Macau and Hainan) and hope that this effort will contribute toward offering insight into the prevention and control of biological invasions in China.

## 2 MATERIALS AND METHODS

### 2.1 Definition

Classifying invasion status of alien species is a controversial practice (e.g., Pyšek, Richardson, & Williamson, 2004). In previous literature, the concepts of alien, exotic, naturalized, and invasive species are often confounded. According to the definition of IUCN, we considered invasive species as those alien species that established in natural ecosystems that threaten native biological diversity, natural environment, economic, and human well‐being (IUCN, 2000; McNeely, Mooney, Neville, Schei, & Waage, 2001; Shine, Williams, & Gundling, 2000). Therefore, only alien species with definitive evidence of negative environmental and social impacts are considered as invasive species here. In our analysis, we focused on the 127 top invasive species reported in Li and Xie (2002), as the invasion status, origins, and life forms of these invaders were well documented.

### 2.2 Data collection

For the top 127 invasive species reported in Li and Xie (2002), 90 of them are plants and 37 are animals. For each of the invasive species, we searched both English and Chinese literature and extracted their landing spots from these references. We gave priority to the primary literature which documented the location where the invasive species was detected for the first time in China. The species entries were supplemented with data on taxonomic position (family), first detection locations, introduction time, and the original references (see Tables S3 and S4). Using this approach, we acquired the data on the first detection locations for 108 of the 127 invasive species in China. The 19 species without known information on their first landing places were deleted from our analysis. Note that our analysis included invasive species that spread naturally as well as those assisted by human activities, such as trade exchanges. Further, we also acquired the trade volumes, population density, area, and GDP: Gross Domestic Product of the 34 provinces and regions of China, to assess the roles of trade and human factors in the distribution and spread of these invasive species.

### 2.3 Data analysis

We first examined the spatial pattern of these top invasive species at the provincial level. For each province or region, we summarized the total number of invasive species and the number of invasive species that were first detected, to determine which province or region is the most common landing place for the invasive species. We further compared the numbers of invasive species that were first detected in Hong Kong to Macao, and Taiwan to Hainan. To see whether the invasive species in mainland China were more likely to come from Hong Kong than its neighboring region—Macao, we used a chi‐square test to compare the observed number of first landed invasive species in Hong Kong and Macao to the null assumption that the number of first landed invasive species in these two regions is evenly distributed. We did a similar analysis for Taiwan and Hainan, to see whether these top invasive species were more likely to first land in Taiwan than Hainan. We used simple linear regression to model the number of top invasive species that first detected in Hong Kong in different provinces and regions, as a function of the geographical distance between these regions and Hong Kong, to assess whether the regions closer to Hong Kong contain more invasive species that first detected in Hong Kong. Further, we also used generalized linear mixed model (GLMM) with logit link and binomial error distribution to assess whether the top invasive species that first detected in Hong Kong have higher chance to present in the regions closer to Hong Kong. To do so, the presence/absence of invasive species first detected in Hong Kong in each region was considered as binary response variable, and the geographical distance of the region to Hong Kong was treated as fixed effects and invasive species identity as random effect. We perform the similar analysis for the invasive species first detected in Taiwan, to see whether these species more likely to present in the regions closer to Taiwan.

To confirm our findings, we also divided these top invasive species into different groups, according to their introduction pathway and taxonomic groups (see Table S1). We firstly divided top invasive plants and animals into intentionally and unintentionally introduced species, respectively. Then, we also divided animal invasive species into three taxonomic groups (insect, fish, and others), and plant invasive species into four taxonomic groups (vine, woody, dry herbs, and aquatic herbs). We perform the similar analysis for each group, to see whether the numbers of first detected invasive species of each group in Hong Kong and Taiwan were larger than Macao and Hainan, respectively (see Table S2). We also used simple linear regression to model the number of first detected invasive species that in different provinces and regions, as a function of the trade volumes, population density, area, and GDP of these regions, to see whether “stepping‐stone” effect relates to the trade and other human factors.

## 3 RESULTS

**Figure 1:**
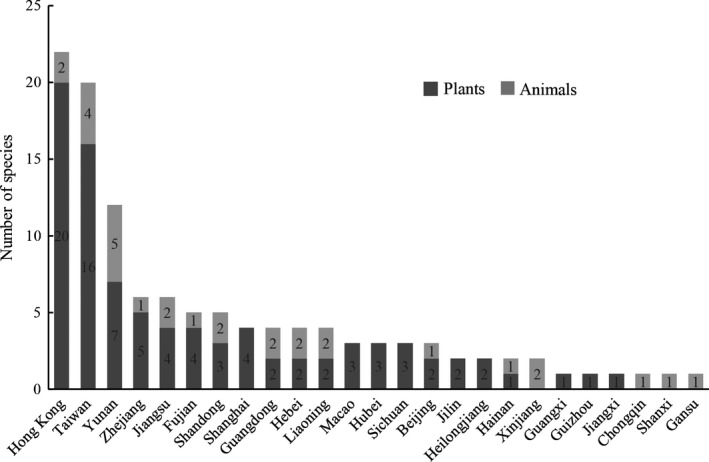
The distribution of first detections of top invasive species across the 34 provinces and regions of China

**Figure 2:**
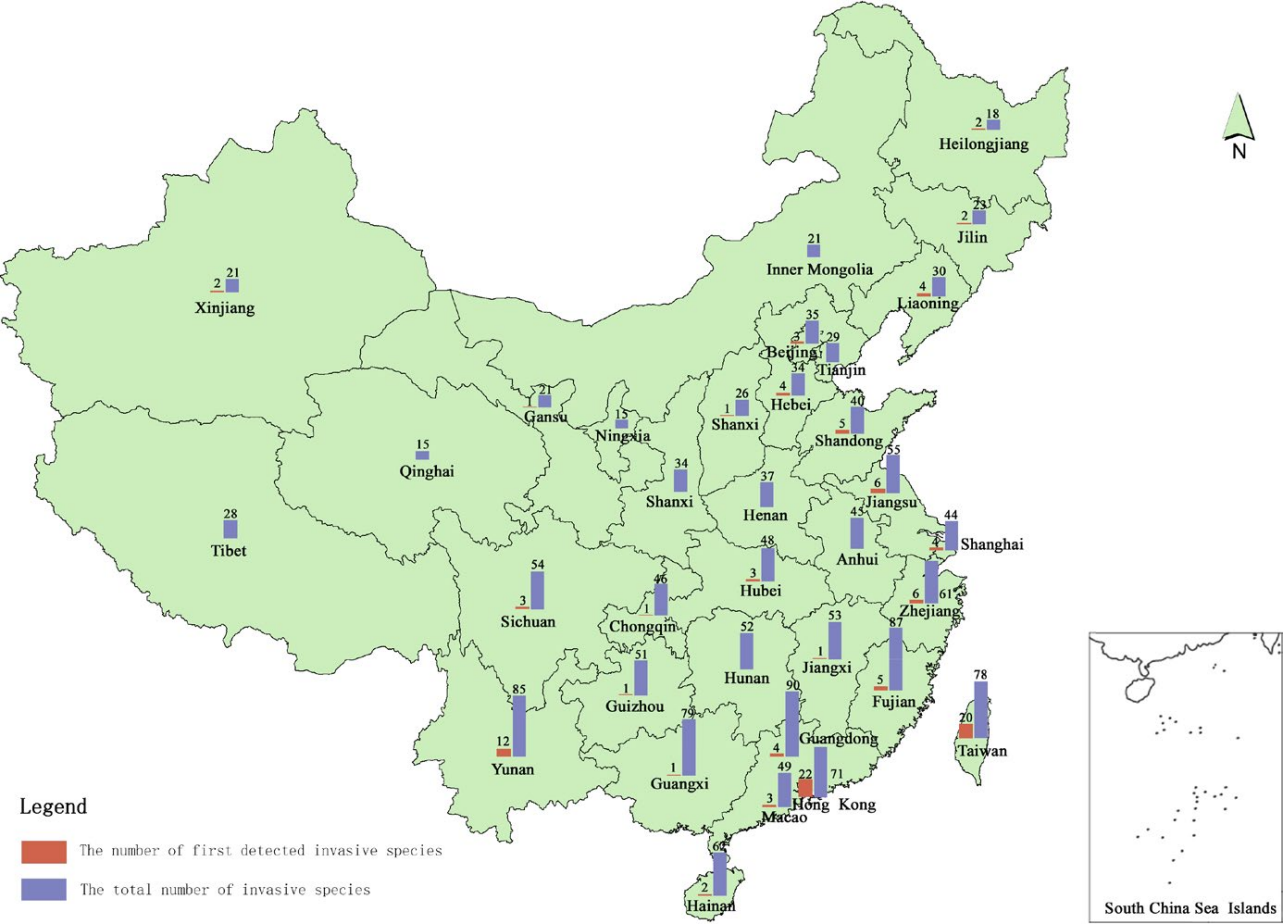
The number of first detections (light red) and the total number (light blue) of invasive species in the 34 provinces and regions of China

The analysis on the landing spots of the top invasive species in China (see Tables S3 and S4) showed that more invasive species were first discovered in Taiwan and Hong Kong than all other provinces or regions of China (see Figure 1). Of the top 127 invasive species, 22 and 20 first settled in Hong Kong and Taiwan, respectively, before spreading to the Chinese mainland. Of the top 90 invasive plants, 36 were first spotted in these two areas, accounting for 40.00% of the total (16 in Taiwan, accounting for 17.78%; 20 in Hong Kong, accounting for 22.22%). Of the 37 top invasive animals, Yunnan Province contains the largest number of first detected invasive animals (five species), following by Taiwan (four species) and Hong Kong (two species). Together, six of 37 (16.22%) top invasive animals were first landed in Taiwan and Hong Kong. Both first detections and the total number of invasive species were mostly distributed in coastal regions and Yunnan province of China (see Figure 2).

By comparing the number of first detected invasive species in Hong Kong and Macao, a nearby region with similar climate conditions and similar colonial history, we found that species in mainland China were more likely to come from Hong Kong than Macau (χ^2^ = 14.44, *p *<* *.001; Table 1). The number of alien species first found in Hong Kong is nearly seven times more than Macau (22 vs. 3 species). Similarly, species in mainland China were also more likely to come from Taiwan than Hainan (χ^2^ = 14.73, *p *<* *.001; Table 1), even though two islands shared similar climate conditions, island areas, and distances to the mainland China. The number of alien species first found in Taiwan is ten times more than Hainan Island (20 vs. 2 species). Top invasive plants are significantly more likely to first land in Hong Kong and Taiwan than Macau and Hainan, respectively (Table 1). Hong Kong and Taiwan also contain more first detected invasive animals than Macau and Hainan, even small numbers of species made the comparison nonsignificant.

**Table 1:** Number of top invasive species first found in Hong Kong, Taiwan, Macao, and Hainan

	Number of species	Percent (%)	χ^2^	*p*
Plant species (90)				
Hong Kong vs. Macao	20 vs. 3	22.22 vs. 3.33	12.57	<.001
Taiwan vs. Hainan	16 vs. 1	17.78 vs. 1.11	13.23	<.001
Animal species (37)				
Hong Kong vs. Macao	2 vs. 0	5.41 vs. 0.00	2.00	.157
Taiwan vs. Hainan	4 vs. 1	10.81 vs. 2.70	1.80	.18
Total (127)				
Hong Kong vs. Macao	22 vs. 3	17.32 vs. 2.36	14.44	<.001
Taiwan vs. Hainan	20 vs. 2	15.75 vs. 1.57	14.73	<.001

**Figure 3:**
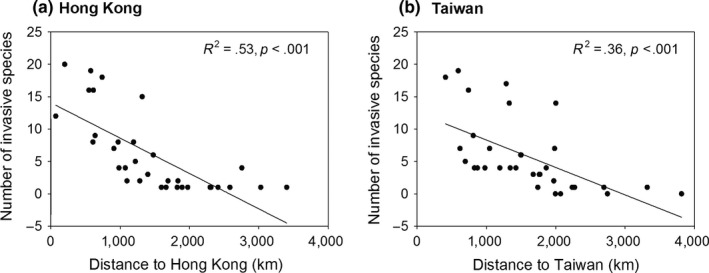
The number of top invasive species, which were first detected in Hong Kong (a) and Taiwan (b), in different provinces and regions, as a function of geographical distance from Hong Kong and Taiwan

We found a positive relationship between the number of top invasive species that first detected in Hong Kong and the region's geographical distance to Hong Kong (*R* ^2^ = .53, *p *<* *.001; Figure 3a), which suggested the regions closer to Hong Kong contained larger number of invasive species first detected in Hong Kong. Further, the GLMMs showed that invasive species first detected in Hong Kong had higher chance to presence in the provinces and regions that nearer to Hong Kong (*p *<* *.001, Table 2). We found similar pattern for the invasive species first detected in Taiwan, which showed bigger quantities and higher presence probabilities in the provinces and regions that closer to Taiwan (*p *<* *.001, Table 2, Figure 3b).

**Table 2:** Results of mixed‐effects models for the presence/absence of invasive species that first detected in Hong Kong or Taiwan in different provinces and regions

	Estimate	Standard error	*z* Value	*p* Value
Distance to Hong Kong	−0.0029	0.0002	−11.5180	<.001
Distance to Taiwan	−0.0020	0.0002	−10.3890	<.001

The analysis on introduction ways of top invasive species in China showed that about one‐third top invasive species were intentional introduction. We found that the number of unintentionally introduced invasive species first found in Hong Kong and Taiwan was higher than Macao and Hainan, respectively (*p* < .10; Table 3). Similarly, the number of intentionally introduced invasive species first found in Taiwan was significantly higher than in Hainan (χ^2^ = 11, *p *=* *.003; Table 3), but the number of intentionally introduced invasive species first found in Hong Kong and Macao did not show significant difference (χ^2^ = 2, *p *=* *.480; Table 3). After we divided all top invasive species into different taxonomic groups, we found that for all the taxonomic groups first detected in the four regions we compared, Hong Kong and Taiwan always contained larger number of first detected top invasive species than Macao and Hainan, respectively (Table S2).

**Table 3:** Number of intentionally and unintentionally introduced invasive species first found in Hong Kong, Taiwan, Macao, and Hainan

	Number of species	Percent (%)	χ^2^	*p*
Intentionally introduced species (40)				
Hong Kong vs. Macao	2 vs. 0	5.00 vs. 0.00	2	.480
Taiwan vs. Hainan	11 vs. 0	27.50 vs. 0.00	11	.003
Unintentionally introduced species (87)				
Hong Kong vs. Macao	20 vs. 3	22.99 vs. 3.45	12.57	<.001
Taiwan vs. Hainan	9 vs. 2	10.35 vs. 2.30	4.45	.070

**Figure 4:**
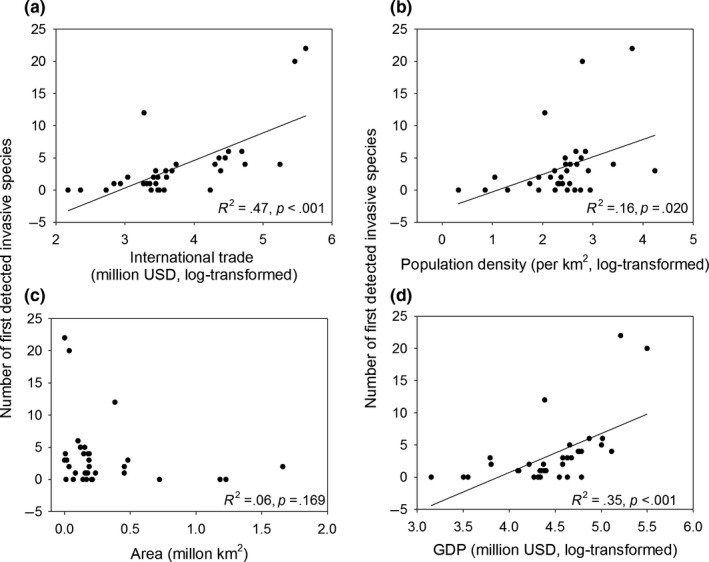
The number of first detections invasive species as a function of trade volumes (a), population density (b), area (c), and GDP (d) of the provinces and regions in year 2000

Our results showed that the number of first detections of invasive species significantly increased with the trade volumes, population density, and GDP of all the regions, whereas area has little effect on the number of first detections invasive species (see Figure 4). Further, we also showed that trade volume was the single best predictor (highest *R* ^2^ value) of the number of first detected invasive species, indicating that international tread may be the key factor in determining which locations become stepping‐stones.

## 4 DISCUSSION

Our study revealed that Hong Kong and Taiwan contain the largest number of first detected top invasive species in China. Therefore, these two areas are two important stepping‐stones for invasive species to the mainland of China. However, what are the reasons for these two areas serving as stepping‐stones? Is it because they both are islands or peninsulas located in the southeastern part of China? To answer this question, we compared the number of first detected invasive species between Hong Kong and Macao, as well as Taiwan and Hainan (see Figure 1).

Macao is a neighboring region of Hong Kong with similar climate conditions and similar colonial history (Figure 2). Hong Kong's land area is 1,104 km^2^ while that of Macao is 29.2 km^2^, and they are only 62 km away from each other (National Bureau of Statistics of China, 2009a). However, the landing and the distribution of the invasive species are strikingly different in these two peninsulas according to our analysis, with Hong Kong holding much more firstly detected invasive species (Table 1). Taiwan is the largest island of China, occupying a land area of 36,000 km^2^, and the minimum distance between the Taiwan Island and the Chinese mainland is only 130 km (Cai & Shi, 2008; National Bureau of Statistics of China, 2009a). Hainan Island, being the second largest island in China, has a land area of 35,000 km^2^ and its minimum distance to the mainland is 19.4 km (National Bureau of Statistics of China, 2009b; Zhao, 2007), being closer to the mainland. The areas of two islands are quite close, and they share similar climate conditions. Their locations are shown in Figure 2. Conceivably, they both could be the stepping‐stone for biological invasions. However, our analysis shows the number of alien species first found in Taiwan is nearly ten times that in Hainan Island (see Table 1). Therefore, it can be concluded that the location, environmental, and climate factors alone could not make Hong Kong and Taiwan the stepping‐stone of invasive species. There are some other factors.

Recent studies suggest that the colonial rule and the related historical events have accelerated biological invasions (Dyer et al., 2017; Wu, Hsieh, Chaw, & Rejmanek, 2004). In fact, Taiwan, as an island of China, was colonized by many different countries in its history. Of the top 16 invasive plant species on the island, six were introduced by different colonial regimes. For example, Wattle (*Acacia farnesiana* W.), White Popinac (*Leucaena leucocephala* W.), and Sweet Prickly Pear (*Opuntia ficus‐indica* M.) were introduced in 1645 when the island was subjected to the rule of the Netherlands. Common Lantana (*Lantana camara* L.) was introduced by the Spanish around the end of the Ming Dynasty (1368‐1644). Johnson Grass (*Sorghum halepense* P.) and Water Hyacinth (*Eichhornia crassipes* S.) were introduced at the beginning of the 20th century by Japanese during their presence on the island (1895–1945) (Li & Xie, 2002; Lu, Wu, Fu, & Zhu, 2007).

However, colonial history also could not fully explain the invasion pattern we found in China. Hong Kong and Macao were both colonized for a long time; Hong Kong was the colony of the Britain, and Macao was the colony of Portugal. It is surprising that the number of firstly detected invasive species in Hong Kong is much more than that in Macao (Table 1). We suggest that trade exchanges would be the driving force for the accumulation and spread of invasive species in Hong Kong. Hong Kong is a world hub for trade, logistics, and transportation, which have proven to be important driving forces for the introduction of alien species (Banks et al., 2015; Perrings et al., 2005; Seebens et al., 2015; Wilson, Dormontt, Prentis, Lowe, & Richardson, 2009; Zhao & Lu, 2007). For example, Slipper Limpet (*Crepidula onyx* S.), the invasive species first found in Hong Kong, was brought from Japan by ocean liners. West Drywood Termite (*Incisitermes minor* H.) was brought to Hong Kong from Japan along with timber and then spread to the Chinese mainland (Ninghai county of Zhejiang province). The fact that most of the alien species in Hong Kong were discovered in wasteland overgrown with weeds also indicates their invasion path associated with import and transportation. Furthermore, the majority of the invasive species in Hong Kong were found around the beginning of the 20th century. A comparison between Taiwan and Hainan shows that the invasive species on the latter island are far fewer owing to the fact that Hainan was not colonized and its trade and economy were less advanced compared with Taiwan. The fact that Hong Kong was a world hub of trade, air, and sea transportation makes the number of invasive species in Hong Kong seven times more than those in Macao. Other studies have also shown that trade plays a key role in the spread of alien species and has arguably contributed to the recent enormous acceleration of biological invasions, thus homogenizing biotas worldwide (Seebens et al., 2015), Dyer et al. (2017) reported that recent introduction of alien bird species in whole world is a wider phenomenon, involving more species and countries, and driven in part by increasing economic activity, the number of introductions to a country in the fourth quartile is positively correlated with its per capita GDP.

Islands or peninsulas are more susceptible to species invasion compared with continents (Gimeno & Hulme, 2006). Our results suggest that different islands and peninsulas may have different roles as stepping‐stones for species invasion in the Chinese mainland because they differ on the colonial rule and international trade. Therefore, to achieve a better management of the invasive species, human activities must be considered to strengthen the management of biological invasion. Firstly, the administration of ports on islands or peninsulas along the coast of Chinese mainland, such as Taiwan, Hainan, Hong Kong, Macao, and Zhoushan archipelago of Zhejiang Province, should be toughened to make it difficult for the invasive species to land in the first place (Ng & Corlett, 2002; Wu et al., 2004; Zhao & Lu, 2007). Secondly, strict animal and plant quarantine should be implemented on any species coming from those areas to the mainland. The introduction of the species that have settled on these islands or peninsulas to the mainland should be minimized to prevent their spreading. Thirdly, based on the geographical characteristics, international trade, and other factors, the islands or peninsulas, should be graded according to their susceptibleness to species invasion, and those areas that are graded as most susceptible should be put under key monitoring, strict control, and management.

Economic and trade exchanges between Hong Kong, Taiwan, and the mainland of China are ever increasing. For example, the mainland China recently allowed the importation of Taiwan's agricultural products, calling for a strict quarantine system to prevent the spread of invasive species into the mainland of China. The Hainan Island, which is striving to become a tourist attraction, is being visited by more international tourists. Minimizing biological invasions to the Hainan Island remains a difficult task.

In 2013, China overtook the United States to become the world's biggest goods trader, with its trade volume accounting for 11% of the world's total. China's exports and imports accounted for 11.7% and 10.3% of the respective world totals, ranking the first and second in the world, respectively. Volume of freight handled at China's coastal ports increased from 310 million tons in 1985 to 7.28 billion tons in 2013, an increase of 22.5 times, to rank first in the world. Therefore, other Chinese cities and regions such as Shanghai, Tianjin, Ningbo (Zhejiang province), and Shenzhen (Guangdong province, near Hong Kong) could rival Hong Kong as a point of entry for many introduced species that will become invasive in the future. We suppose that they would be the future stepping‐stones for invasive species to the mainland of China and these cities where improved biosecurity is needed now.

Dawson et al. (2017) had assessed global patterns and potential drivers of established alien species richness across all taxonomic groups, their results highlighted the need to prioritize prevention of further alien species introduction to island and coastal mainland regions globally. It is important to use complementary approaches, such as reconstructing routes of invasion using genetic data, to fully understand the spread pathways and stepping‐stones of invasive species.

## CONFLICT OF INTEREST

None declared.

## AUTHOR CONTRIBUTIONS

J. L. and L. J. conceived the ideas and led the writing. S. L. and J. L. performed the analyses. Y. W. and J. L. collected the data. L. J. improved the ideas and analyses. S. L. and J. L. revised the manuscript. All authors gave the final approval for publication.
