# Supplementary material for: Are Hong Kong and Taiwan stepping‐stones for invasive species to the mainland of China?
Source: Ecol Evol. 2018 Jan 15;8(4):1966–73. doi: 10.1002/ece3.3818 (PMC5817137; doi:10.1002/ece3.3818)
Supplement: Supplementary file 1 [file ECE3-8-1966-s001.doc]

**Supporting Information**

**Table S1.** Introduction ways of top invasive species in different taxonomic groups in China.

| Taxonomic groups | Animal | | |  | Plant | | | |
| --- | --- | --- | --- | --- | --- | --- | --- | --- |
|  | Insect | Fish | Others |  | Vine | Woody | Dry herbs | Aquatic herbs |
| Intentional introduction | 0 | 5 | 6 |  | 3 | 7 | 15 | 4 |
| Unintentional introduction | 20 | 2 | 4 |  | 3 | 3 | 54 | 1 |
| Total | 20 | 7 | 10 |  | 6 | 10 | 69 | 5 |

**Table S2.** Number of top invasive species first found in Hong Kong, Taiwan, Macao and Hainan, separated by taxonomic groups.

|  |  | Number of species | Percent (%) |
| --- | --- | --- | --- |
| **Insect Species (20)** | |  |  |
|  | Hong Kong vs. Macao | 1 vs. 0 | 5.00 vs. 0 |
|  | Taiwan vs. Hainan | 2 vs. 1 | 10.00 vs. 5.00 |
|  |  |  |  |
| **Fish Species (7)** | |  |  |
|  | Hong Kong vs. Macao | 0 vs. 0 | 0 vs. 0 |
|  | Taiwan vs. Hainan | 0 vs. 0 | 0 vs. 0 |
|  |  |  |  |
| **Other Animals Species (10)** | |  |  |
|  | Hong Kong vs. Macao | 1 vs. 0 | 10.00 vs. 0 |
|  | Taiwan vs. Hainan | 2 vs. 0 | 20.00 vs. 0 |
|  |  |  |  |
| **Vine Species (6)** | |  |  |
|  | Hong Kong vs. Macao | 3 vs. 0 | 50.00 vs. 0 |
|  | Taiwan vs. Hainan | 1 vs. 0 | 16.67 vs. 0 |
|  |  |  |  |
| **Woody Species (10)** | |  |  |
|  | Hong Kong vs. Macao | 1 vs. 1 | 10.00 vs. 10.00 |
|  | Taiwan vs. Hainan | 4 vs. 0 | 40.00 vs. 0 |
|  |  |  |  |
| **Dry Herbs** **Species (69)** | |  |  |
|  | Hong Kong vs. Macao | 16 vs. 2 | 23.19 vs. 2.90 |
|  | Taiwan vs. Hainan | 10 vs. 1 | 14.49 vs. 1.45 |
|  |  |  |  |
| **Aquatic Herbs Species (5)** | |  |  |
|  | Hong Kong vs. Macao | 0 vs. 0 | 0 vs. 0 |
|  | Taiwan vs. Hainan | 1 vs. 0 | 20.00 vs. 0 |

**Table S3. Listing of top invasive plant species in China.**

| ID | Scientific Name | Taxonomic Group | Family | First Found in | Introduction Way | Introduction Time | References |
| --- | --- | --- | --- | --- | --- | --- | --- |
| 1 | *Acacia farnesiana* W. | Woody | Leguminosae | Taiwan | Intentional | 1645 | (Li & Xie, 2002) |
| 2 | *Acanthospermum australe* K. | Dry herbs | Compositae | Yunnan | Unintentional | 1936 | (Li & Xie, 2002) |
| 3 | *Ageratum conyzoides* L. | Dry herbs | Compositae | Hong Kong | Unintentional | 19th century | (Bentham, 1861) |
| 4 | *Agrostemma githago* L. | Dry herbs | Caryophyllaceae | Northeast | Unintentional | 19th century | (Xu & Qiang. 2004) |
| 5 | *Alternanthera philoxeroides* G. | Aquatic herbs | Amaranthaceae | Shanghai | Intentional | 1892 | (Li & Xie, 2002) |
| 6 | *Alternanthera pungens* H. B. K. | Dry herbs | Amaranthaceae | Sichuan | Unintentional | 1957 | (Li & Xie, 2002) |
| 7 | *Amaranthus polygonoides* L. | Dry herbs | Amaranthaceae | Shandong | Unintentional | 1979 | (Li & Xie, 2002) |
| 8 | *Amaranthus retroflexus* L. | Dry herbs | Amaranthaceae | Hebei & Shandong | Unintentional | 1850 | (Li & Xie, 2002) |
| 9 | *Amaranthus spinosus* L. | Dry herbs | Amaranthaceae | Macao | Unintentional | 1830 | (Xu & Qiang 2004) |
| 10 | *Amaranthus viridis* L. | Dry herbs | Amaranthaceae | Taiwan | Unintentional | 1864 | (Xu & Qiang 2004) |
| 11 | *Ambrosia artemisiifolia* L. | Dry herbs | Compositae | Zhejiang | Unintentional | 1935 | (Li & Xie, 2002) |
| 12 | *Anredera cordifolia* S. | Vine | Basellaceae | Taiwan | Intentional | 1976 | (Xu, Sheng, Genovesi, Hui, Wu, & Ling 2012) |
| 13 | *Apium leptophyllum* F. J. M. | Dry herbs | Umbelliferae | Hong Kong | Unintentional | Beginning of 20th century | (Dunn and Tutcher, 1912) |
| 14 | *Aster subulatus* M. | Dry herbs | Compositae | Hubei | Unintentional | 1947 | (Li & Xie, 2002) |
| 15 | *Avena fatua* L. | Dry herbs | Gramineae | Hong Kong & Fujiang | Unintentional | Middle of 19th century | (Xu, Sheng, Genovesi, Hui, Wu, & Ling 2012) |
| 16 | *Axonopus compressus* B. | Dry herbs | Gramineae | Taiwan | Intentional | 1940 | (Li & Xie, 2002) |
| 17 | *Bidens pilosa* L. | Dry herbs | Compositae | Hong Kong | Unintentional | 1857 | (Seemann, 1857) |
| 18 | *Cabomba caroliniana* G. | Aquatic herbs | Nymphaeaceae | Zhejiang | Unintentional | 1993 | (Li & Xie, 2002) |
| 19 | *Cenchrus echinatus* L. | Dry herbs | Gramineae | Taiwan | Unintentional | 1934 | (Li & Xie, 2002) |
| 20 | *Chenopodium ambrosioides* L. | Dry herbs | Chenopodiaceae | Taiwan | Unintentional | 1864 | (Li & Xie, 2002) |
| 21 | *Chenopodium hybridum* L. | Dry herbs | Chenopodiaceae | Hebei | Unintentional | 1864 | (Li & Xie, 2002) |
| 22 | *Conyza canadensis* C. | Dry herbs | Compositae | Shandong | Unintentional | 1860 | (Li & Xie, 2002) |
| 23 | *Conyza sumatrensis* W. | Dry herbs | Compositae | Unknown | Unintentional | Middle of 19th century | (Li & Xie, 2002) |
| 24 | *Coronopus didymus* J. E. S. | Dry herbs | Cruciferae | Jiangsu | Unintentional | 1930 | (Li & Xie, 2002) |
| 25 | *Crassocephalum crepidioides* S. M. | Dry herbs | Compositae | Guangxi | Unintentional | 1930s | (Li & Xie, 2002) |
| 26 | *Datura stramonium* L. | Dry herbs | Solanaceae | Unknown | Intentional | Late Ming Dynasty | (Li & Xie, 2002) |
| 27 | *Daucus carota* L. | Dry herbs | Umbelliferae | Unknown | Unintentional | 1406 | (Li & Xie, 2002) |
| 28 | *Eichhornia crassipes* S. | Aquatic herbs | Pontederiaceae | Taiwan | Intentional | 1901 | (Li & Xie, 2002) |
| 29 | *Erigeron annuus* P. | Dry herbs | Compositae | Shanghai | Unintentional | 1886 | (Forbes & Hemsley, 1890) |
| 30 | *Eryngium foetidum* L. | Dry herbs | Umbelliferae | Yunnan | Intentional | 1897 | (Li & Xie, 2002) |
| 31 | *Eupatorium adenophorum* S. | Dry herbs | Compositae | Yunnan | Unintentional | 1935 | (Li & Xie, 2002) |
| 32 | *Eupatorium catarium* V. | Dry herbs | Compositae | Hong Kong | Unintentional | 1980s | (Li & Xie, 2002) |
| 33 | *Eupatorium odoratum* L. | Dry herbs | Compositae | Yunnan | Unintentional | 1934 | (Gagnepain, 1924) |
| 34 | *Euphorbia dentata* M. | Dry herbs | Euphorbiaceae | Beijing | Unintentional | 1976 | (Li & Xie, 2002) |
| 35 | *Euphorbia hirta* L. | Dry herbs | Euphorbiaceae | Macao | Unintentional | 1820 | (Li & Xie, 2002) |
| 36 | *Euphorbia maculata* L. | Dry herbs | Euphorbiaceae | Shanghai & Jiangsu | Unintentional | 1940 | (Li & Xie, 2002) |
| 37 | *Galinsoga parviflora* C. | Dry herbs | Compositae | Yunnan | Unintentional | 1915 | (Handel-Mazzetti, 1936) |
| 38 | *Geranium caroliniamum* L. | Dry herbs | Geraniaceae | Jiangsu | Unintentional | 1926 | (Xu, Sheng, Genovesi, Hui, Wu, & Ling 2012) |
| 39 | *Hibiscus trionum* L. | Dry herbs | Malvaceae | Unknown | Unintentional | 1406 | (Li & Xie, 2002) |
| 40 | *Hyptis rhomboidea* M. G. | Dry herbs | Labiatae | Hainan | Unintentional | 1922 | (Li & Xie, 2002) |
| 41 | *Hyptis suaveolens* P. | Dry herbs | Labiatae | Taiwan | Unintentional | End of 19th century | (Li & Xie, 2002) |
| 42 | *Ipomoea cairica* S. | Vine | Convolvulaceae | Hong Kong | Unintentional | 1912 | (Li & Xie, 2002) |
| 43 | *Ipomoea purpurea* R. | Vine | Convolvulaceae | Unknown | Intentional | 1980 | (Forbes & Hemsley, 1890) |
| 44 | *Lantana camara* L. | Woody | Verbenaceae | Taiwan | Intentional | 1645 | (Li & Xie, 2002) |
| 45 | *Lepidium virginicum* L. | Dry herbs | Cruciferae | Hubei | Unintentional | 1933 | (Li & Xie, 2002) |
| 46 | *Leucaena leucocephala* W. | Woody | Leguminosae | Taiwan | Intentional | 1645 | (Li & Xie, 2002) |
| 47 | *Lolium temulentum* L. | Dry herbs | Gramineae | Unknown | Unintentional | 1940s | (Li, 1998) |
| 48 | *Macfadyena unguis-cati* A. G. | Vine | Bignoniaceae | Fujian | Intentional | 1840 | (Li & Xie, 2002) |
| 49 | *Malvastrum coromandelianum* G. | Dry herbs | Malvaceae | Hong Kong | Unintentional | 1861 | (Li & Xie, 2002) |
| 50 | *Mikania micrantha* H. B. K. | Vine | Compositae | Hong Kong | Unintentional | 1919 | (IUCN, 2001) |
| 51 | *Mimosa pudica* L. | Dry herbs | Leguminosae | Unknown | Intentional | Late Ming Dynasty | (IUCN, 2001) |
| 52 | *Mirabilis jalapa* L. | Dry herbs | Nyctaginaceae | Zhejiang | Intentional | 16th century | (Xu, Sheng, Genovesi, Hui, Wu, & Ling 2012) |
| 53 | *Opuntia ficus-indica* M. | Woody | Cactaceae | Taiwan | Intentional | 1645 | (Li & Xie, 2002) |
| 54 | *Opuntia monacantha* H. | Woody | Cactaceae | Yunnan | Intentional | 1625 | (Li & Xie, 2002) |
| 55 | *Opuntia stricta* H. B. | Woody | Cactaceae | Unknown | Intentional | Late Ming Dynasty | (Li & Xie, 2002) |
| 56 | *Oxalis corymbosa* DC. | Dry herbs | Oxalidaceae | Hong Kong | Intentional | 1850 | (Bentham, 1861) |
| 57 | *Panicum maximum* J. | Dry herbs | Gramineae | Taiwan | Intentional | 1908 | (Dunn |
|  |  |  |  |  |  |  | & Tutcher, 1912; Ng |
|  |  |  |  |  |  |  | & Richard, 2002) |
| 58 | *Panicum repens* L. | Dry herbs | Gramineae | Hong Kong | Unintentional | 1857 | (Li & Xie, 2002) |
| 59 | *Parthenium hysterophorus* L. | Dry herbs | Compositae | Yunnan | Unintentional | 1926 | (Gagnepain, 1924) |
| 60 | *Paspalum conjugatum* B. | Dry herbs | Gramineae | Hong Kong | Unintentional | 1912 | (Dunn & Tutcher, 1912) |
| 61 | *Passiflora foetida* L. | Vine | Passifloraceae | Hong Kong | Unintentional | 1861 | (Bentham, 1861) |
| 62 | *Peperomia pellucida* H. B. K. | Dry herbs | Piperaceae | Hong Kong | Unintentional | Beginning of 20th century | (Dunn & Tutcher, 1912) |
| 63 | *Phytolacca americana* L. | Dry herbs | Phytolaccaceae | Zhejiang | Intentional | 1935 | (Li & Xie, 2002) |
| 64 | *Pilea microphylla* L. | Dry herbs | Urticaceae | Taiwan | Unintentional | 1928 | (Li & Xie, 2002) |
| 65 | *Pistia stratiotes* L. | Aquatic herbs | Araceae | Unknown | Intentional | Late Ming Dynasty | (Li & Xie, 2002) |
| 66 | *Plantago virginica* L. | Dry herbs | Plantaginaceae | Jiangxi | Unintentional | 1951 | (Li & Xie, 2002) |
| 67 | *Rhynchelytrum repens* H. | Dry herbs | Gramineae | Taiwan | Intentional | 1950s | (Li & Xie, 2002) |
| 68 | *Ricinus communis* L. | Dry herbs | Euphorbiaceae | Unknown | Intentional | 659 | (Li & Xie, 2002) |
| 69 | *Scoparia dulcis* L. | Dry herbs | Scrophulariaceae | Hong Kong | Unintentional | Middle of 19th century | (Bentham, 1861) |
| 70 | *Senecio vulgaris* L. | Dry herbs | Compositae | Northeast | Unintentional | 19th century | (Dunn & Tutcher, 1912) |
| 71 | *Solanum aculeatissimum* J. | Dry herbs | Solanaceae | Guizhou | Unintentional | End of 19th century | (Li & Xie, 2002) |
| 72 | *Solanum capsicoides* A. | Dry herbs | Solanaceae | Hong Kong | Unintentional | 1895 | (Li & Xie, 2002) |
| 73 | *Solanum erianthum* D. D. | Woody | Solanaceae | Fujian | Unintentional | 1857 | (Li & Xie, 2002) |
| 74 | *Solanum torvum* S. | Woody | Solanaceae | Macao | Unintentional | 1827 | (Li & Xie, 2002) |
| 75 | *Solidago canadensis* L. | Dry herbs | Compositae | Shanghai | Intentional | 1935 | (Xu, Sheng, Genovesi, Hui, Wu, & Ling 2012) |
| 76 | *Soliva anthemifolia* R. B. | Dry herbs | Compositae | Hong Kong | Unintentional | 1912 | (Dunn & Tutcher, 1912) |
| 77 | *Sorghum halepense* P. | Dry herbs | Gramineae | Taiwan | Intentional | Beginning of 20th century | (Dunn & Tutcher, 1912) |
| 78 | *Spartina alterniflora* L. | Aquatic herbs | Gramineae | Fujian | Intentional | 1979 | (Xu, Sheng, Genovesi, Hui, Wu, & Ling 2012) |
| 79 | *Spermacoce latifolia* K. S. | Dry herbs | Rubiaceae | Guangdong | Intentional | 1937 | (Li & Xie, 2002) |
| 80 | *Stachytarpheta jamaicensis* V. | Dry herbs | Verbenaceae | Hong Kong | Unintentional | End of 19th century | (Dunn & Tutcher, 1912; Forbes & Hemsley, 1890) |
| 81 | *Synedrella nodiflora* G. | Dry herbs | Compositae | Hong Kong | Unintentional | 1912 | (Dunn & Tutcher, 1912) |
| 82 | *Tridax procumbens* L. | Dry herbs | Compositae | Taiwan | Unintentional | 1933 | (Li & Xie, 2002) |
| 83 | *Ulex europaeus* L. | Woody | Leguminosae | Sichuan | Intentional | 1862 | (IUCN, 2001) |
| 84 | *Veronica hederaefolia* L. | Dry herbs | Scrophulariaceae | Zhejiang & Jiangsu | Unintentional | 1980s | (Guo & Liu, 2001) |
| 85 | *Veronica persica* P. | Dry herbs | Scrophulariaceae | Hubei | Unintentional | 1933 | (Li & Xie, 2002) |
| 86 | *Veronica polita* P. | Dry herbs | Scrophulariaceae | Unknown | Unintentional | 1406 | (Li, 1998) |
| 87 | *Vetiveria zizanioides* L. | Dry herbs | Gramineae | Guangdong | Intentional | 1920s | (Hitchcock, 1929) |
| 88 | *Waltheria indica* L. | Woody | Sterculiaceae | Hong Kong | Unintentional | 1861 | (Bentham, 1861) |
| 89 | *Wedelia trilobata* H. | Dry herbs | Compositae | Hong Kong | Intentional | 1976 | (IUCN, 2001) |
| 90 | *Xanthium spinosum* L. | Dry herbs | Compositae | Beijing | Unintentional | 1974 | (Li & Xie, 2002) |

**Table S4.** Listing of top invasive animal species in China.

| ID | Scientific Name | Taxonomic Group | Family | First Found in | Introduction Way | Introduction Time | References |
| --- | --- | --- | --- | --- | --- | --- | --- |
| 91 | *Achatina fulica* F. | Others | Achatindae | Fujian | Intentional | 1920s | (Chen, Zhang, Zhang, Zheng & Cheng, 1996; APQB, 1999; Zhou, Chen, Cai & Li, 1989; Chen & Deniu, 1991) |
| 92 | *Anoplolepis gracilipes* S. | Insect | Formicidae | Unknown | Unintentional | Unknown | (Fowler, Schlindwein, & Medeiros, 1994; Haines & Haines, 1978; Wheeler, 1910) |
| 93 | *Aristichthys nobilis* R. | Fish | Cyprinidae | Yunnan | Intentional | 1950s-1960s | (Chen, 1998; Chen, Yang, & Li, 1998; Chen, Yang, Su, & Chen, 2001; He, 1998; Zhang, Cao, & Chen, 1997) |
| 94 | *Bruchus pisorum* L. | Insect | Bruchidae | Heibei | Unintentional | 1950s | (NATESC, 1998; Zhang, Liu, & Wu, 1998; APQB, 1999) |
| 95 | *Bruchus rufimanus* B. | Insect | Bruchidae | Unknown | Unintentional | 1930s | (NATESC, 1998; Zhang, Liu, & Wu, 1998; APQB, 1999) |
| 96 | *Bursaphelenchus xylophilus* S. & B. | Others | Aphelechoididae | Jiangsu | Unintentional | 1982 | (Ding & Xie, 2001; Wang, 1992) |
| 97 | *Callosobruchus maculatus* F. | Insect | Bruchidae | Taiwan | Unintentional | Unknown | (NATESC, 1998; Zhang, Liu, & Wu, 1998; APQB, 1999) |
| 98 | *Crepidula onyx* S. | Others | Calyptraeidae | Hong Kong | Unintentional | 1979 | (Huang, Brian, & Ye, 1984; Huang, 1984; Huang, Morton, & Yipp, 1983) |
| 99 | *Ctenopharyngodon idellus* C. & V. | Fish | Cyprinidae | Yunnan | Intentional | 1950s-1960s | (Chen, 1998; Chen, Yang, & Li, 1998; Chen, Yang, Su, & Chen, 2001; He, 1998; Liu, & Qin, 1987; Yang, 2001; Zhang, Cao, & Chen, 1997; Zhu, 1995) |
| 100 | *Dendroctonus valens* L. | Insect | Scolytidae | Shanxi | Unintentional | 1998 | (Chang, Liu, Zhao, Sun, Ma, & Xu, 2001; Wu, 2000; Yin, 2000; Eaton & Lara, 1967) |
| 101 | *Eriosoma lanigerum* H. | Insect | Pemphigidae | Shandong & Liaoning | Unintentional | 1914 | (Feng, 1992; NATESC, 1998; Zhang & Zhao, 1996; APQB, 1999) |
| 102 | *Gambusia affinis* B. & G. | Fish | Poecillidae | Zhejiang | Intentional | 1924 | (Zhu, 1995; Lever, 1997; Tan & Tong, 1989) |
| 103 | *Hemiberlesia pitysophila* T. | Insect | Diaspididae | Taiwan | Unintentional | 1965 | (He, 1995; Wang, 1992; Zhang & He, 1997; APQB, 1999) |
| 104 | *Hyphantria cunea* D. | Insect | Arctiidae | Liaoning | Unintentional | 1979 | (Ding, 2001; Feng, 1992; Wang, 1992; Wang, 1996; Zhang & He, 1997; APQB, 1999) |
| 105 | *Incisitermes minor* H. | Insect | Kalotermitidae | Hong Kong | Unintentional | 1937 | (He, Huang, Jing, & Gao, 1992,; Huang, Zhang, & Tao, 1992; Liu, Jiang, Su, Peng, Wei, & Shi, 1998) |
| 106 | *Laspeyresi pomonella* L. | Insect | Torticidae | Gansu | Unintentional | 1987 | (Feng, 1992; NATESC, 1998; Zhang, & He, 1997; APQB, 1999) |
| 107 | *Lehmannia valentiana* F. | Others | Limacidae | Unknown | Unintentional | Unknown | (Wiktor, Chen, & Wu, 2000) |
| 108 | *Leptinotarsa decemlineata* S. | Insect | Chrsomelidae | Xinjiang | Unintentional | 1992 | (Cheng & Ma, 2000; Wang & Ding, 1996; Wu, 2000; Zhang & He, 1997; APQB, 1999) |
| 109 | *Liriomyza sativae* B. | Insect | Agromyzidae | Hainan | Unintentional | 1993 | (Cheng & Xiao, 1995; Ding & Xie, 2001; NATESC, 1998; Tan & Zhuo, 1997) |
| 110 | *Lissorhoptrus oryzophilus* K. | Insect | Curculionidae | Heibei | Unintentional | 1988 | (Cai et al., 1997; NATESC, 1998; Zhang, Yu, & Xu, 1994; APQB, 1999) |
| 111 | *Myocastor coypus* M. | Others | Myocastoridae | Unknown | Intentional | 1953 | (Huang, Cheng, & Wen, 1995; Xu, Ding, & Zeng, 1997; Zou, & Wang, 1997) |
| 112 | *Mytilopsis sallei* R. | Others | Dreissenidae | Taiwan | Unintentional | 1977 | (Wang, Huang, Zheng, & Lin, 1999; Zhang, 1985; Huang & Morton, 1993) |
| 113 | *Neosalanx taihuensis* C. | Fish | Salangidae | Yunnan | Intentional | 1979 | (Cheng, Yang, & Li, 1998; Cheng, Yang, & Su, 2001; Yang, 2001; Zhang, Cao, & Cheng, 1997; Zhu, 1995; Liu, Zhu, & Wang, 1995) |
| 114 | *Ondatra zibethica* L. | Others | Muridae | Xinjiang | Intentional | 1950 | (Cheng, 1986; Huang, Cheng, & Wen, 1995; Ma, & Lou, 1980; Ma, 1997) |
| 115 | *Opogona sacchari* B. | Insect | Hieroxestidae | Guangdong | Unintentional | 1987 | (Cheng & Yang, 1997; Cheng & Yang, 1997; Cheng, Lu, & Yang, 1998; Feng & Chen, 1999) |
| 116 | *Oracella acuta* L. | Insect | Pseudococcidae | Guangdong | Unintentional | 1988 | (He, 1995; Pang & Tang, 1994; Yang, 1993; Zhang & He, 1997) |
| 117 | *Oreochromis spp.* G. | Fish | Cichlidae | Unknown | Intentional | 1957 | (Liu, & Qin, 1987; Wang, 2001; Lever, 1997; Tan & Tong, 1989) |
| 118 | *Periplaeta americana* L. | Insect | Blattidae | Unknown | Unintentional | Unknown | (Wu, 1982) |
| 119 | *Pheidole megacephala* F. | Insect | Formicidae | Unknown | Unintentional | Unknown | (Fellowes, 1999; Hoffmann, Andersen, & Hill, 1999; Kadoorie & Botanic, 2002; Reimer, 1994) |
| 120 | *Pomacea canaliculata* S. | Others | Ampullariidae | Taiwan | Intentional | 1970s | (Cai & Chen, 1990; Ding & Xie, 2001; Feng, 1994; Naylor, 1996) |
| 121 | *Procambius clarkii* G. | Others | Cambaridae | Jiangsu | Intentional | 1929 | (Yao, Sun, Guo, & Lin, 1995; Zheng, 1999) |
| 122 | *Pseudorasbora parva* T. & S. | Fish | Cyprinidae | Yunnan | Unintentional | 1960s-1970s | (Chen, 1998; Chen, Yang, & Li, 1998; Chen, Yang, & Su, 2001; He, 1998; Liu, & Qin, 1987; Wang, 2001; Yang, 2001; Zhang, Cao, & Chen, 1997; Zhu, 1995) |
| 123 | *Rana catesbeiana* S. | Others | Ranidae | Beijing | Intentional | 1959 | (Chen, Yang, & Li, 1998; Chen, Yang, & Su, 2001; He, 1998; Li & Wang, 1995; Ye, Fei, & Hu,1993) |
| 124 | *Rhinogobius giurinus* R. | Fish | Gobiidae | Yunnan | Unintentional | 1960s-1970s | (Chen, Yang, & Li, 1998; Chen, Yang, & Su, 2001; He, 1998; Liu, & Qin, 1987; Wang, 2001; Yang, 2001; Zhang, Cao, & Chen, 1997; Zhu, 1995) |
| 125 | *Trogoderma granarium* E. | Insect | Dermestidae | Unknown | Unintentional | 1962 | (NATESC, 1998; Wu, 2000; Zhang, Liu, & Wu, 1998; APQB, 1999) |
| 126 | *Viteus vitifoliae* F. | Insect | Phylloxeridae | Shandong | Unintentional | 1895 | (Feng, 1992; NATESC, 1998; Zhang & Zhao, 1996; APQB, 1999) |
| 127 | *Zabrotes subfasciatus* B. | Insect | Bruchidae | Chongqing | Unintentional | 1987 | (NATESC, 1998; Zhang, Liu, & Wu, 1998; APQB, 1999) |

**REFERENCES**

Bentham, G. (1861). Flora Hongkongensis: A Description of the Flowering Plants and Ferns. London: Lovell Reeve.

Cai, H. X. & Cheng, R. Z. (1990). New pests -- *Pomacea canaliculata*. *Guangdong Agricultural Sciences, (5):* 36~38.

Cai, Y., Wang, X., Cheng, H. C., Liu, S. S., Gao, B. Z., & Song, F. (1997). The study of *Lissorbqptrus oryzqphilus Kuschel* propagation path (1). *Plant Quarantine, 11 (1):* 38~40.

Chang, B. S., Liu, S. C., Zhao, X. M., Sun, Y. M., Ma, F., & Xu, T. X. (2001). The study of occurrence rule of *Dendroctonus valens LeConte*. *Shanxi Forestry Science and Technology, (4):* 1~4.

Chen, D. N., Zhang, G. Q., Zhang, G. Cheng, Y. X., & Zhang, W. (1996). The African giant snail spreads its territory in Yunnan. *Plant Quarantine, (1):* 12~13.

Chen, Deniu, (1991). A study on *Achatina fulica* (FER.) in China (classification, distubution and artificial breeding). *Pro. Tenth Intern. Malacol. Congr.* (Tubingen 1989) 297~301.

Chen, L. Z. & Ma, K. P. (2000). Biodiversity Science: Principle and Practice. Shanghai: Shanghai Science and Technology Press.

Chen, Y. R., Yang, J. X., & Li, Z. Y. (1998). The diversity and present status of fishesin Yunnan Province. *Biodiversity Science, 6 (4):* 272~227.

Chen, Y. Y.(1998). Fauna Sinica · Osteichthyes Cypriniformes (In volume). Beijing: Science Press.

Chen, Z. M., Yang, J. X., Su, R. F., & Cheng, X. Y. (2001). Present status of the indigenous f ishes in Dianchi Lake , Yunnan. *Biodiversity Science, 9 (4):* 407~413.

Cheng, D. S. (1986). A brief analysis of the origin Xingjiang Ondatra zibethicus. *Wild Animal, 7 (5):* 52.

Cheng, G. F. & Yang, J. K. (1997). The quarantine new insect found in Beijing -- *Opogona sacchari* first report. *Plant Quarantine, 11 (2):* 95~101.

Cheng, G. F. & Yang, J. K. (1997). The situation of *Opogona sacchari* in our country. *Plant Protection, 23 (6):* 46~47.

Cheng, G. F., Lu, Q., & Yang, J. K. (1998). The reasons of severe occurrence about *Opogona sacchari* and prevention countermeasures. *Plant Quarantine, 12 (2):* 95~97.

Cheng, N. Z. & Xiao, L. (1995). The spread and harmful of *Liriomyza bryoniae*. *Plant Quarantine, 9 (1):* 6~9.

Ding, J. Q. & Xie, Y. (2001). The invasive mechanism and countermeasure of alien species in China. View: *China Biodiversity Conservation (2)*. Beijing: China Environmental Science Press.

Dunn, S. T. & W. T. Tutcher. (1912). Flora of Kwangtung and Hongkong (China). *Bulletin of Miscellaneous Information, Additional Series 10:* 1~370.

Eaton, C. B., & Lara, R. R. (1967). Red turpentine beetle *Dendroctonus valens LeConte*. In: A.G. Davidson and R. M. Prentice (eds.). Important Forest Insects of Mutual Concern to Canada, the United States and Mexico. Canada Department of Forestry and Rural Development Pub. 1180. Ottawa, 248 pp.

Fellowes, J. R. (1999). Exotic ants in Asia: is the mainland at risk? The case of Hong Kong. *Aliens 9:* 5~6.

Feng, S. X. & Chen, N. Z. (1999). Quarantine the pest of *Opogona sacchari.* *Plant Quarantine, 13 (3):* 42~43.

Feng, W. M. (1994). The biological characteristics and prevention countermeasures of *Pomacea canaliculata*. *Guangdong Agricultural Science, (6):* 41~43.

Forbes, F. B. & W. B. Hemsley. (1890). Enumeration of all the plants known from China Poper, Formosa, Hainan, the Corea, the Luchu Archipelago, and the island of Hongkong. *Journal of Linnear Society of Botany, 26:* 1~316.

Fowler, H. G., Schlindwein, M. N. & de Medeiros, M. A., (1994). Exotic ants and community simplification in Brazil: a review of the impact of exotic ants on native ant assemblages. Pp. 151~162 in Williams DF.

Gagnepain, F. (1924). Composees. In Lecomte H (ed.). Flore Generale L’Indo-Chine. Paris: Masson & Cie. Editeurs, 3: 448~663.

Guo, S. L. & Liu, X. Z. (2001). The chronosome number of *Veronica hederaefolia L*. in China and its ecological significance. *Guihaia, 21 (2):* 111~112.

Haines, I. H. & Hanies, J. B. (1978). Pest status of the crazy ant, *Anoplolepis longipes* (Jerdon) (Hymenoptera: Formicidae), in the Seychelles. *Bulletin of Entomological Research 68:* 627~638.

Handel-Mazzetti, H. (1936). Symbolae Sinicae. Wien: Verlag von Julius Springer.

He, C. H., Huang, Z. C., Jin, Y. L., & Gao, Y. M. (1992). The new mulberry filed pest in Huzhou area -- *Odontotermesformosanus*. *Bulletin of Sericulture, 23 (2):* 34~35.

He, G. F. (1995). The introduction and control situation of two risk forest insects in Guangdong province. *Plant Protection, 21 (2):* 47~48.

He, X. R. (1998). *Hypselotriton wolterstorffi*, an analysis of the Factors caused its extinction. *Sichuan Journal of Zoology, 17 (2):* 58~60.

Hitchcock, A.S. 1929. Grasses of Canton and Vicinity. *Lingnan Science Journal, 7:* 177~265.

Hoffmann, B. F., Andersen, A. N., & Hill, G.J.E. (1999). Impact of an introduced ant on native rain forest invertebrates: *Pheidole megacephala* in monsoonal Australia. *Oecologia 120:* 595~604.

Huang, B. Y., Zhang, X. Y., & Tao, W. Q. (1992). Investigation on the species distributions and harmfulness of termite in Hubei province. *Journal of Huazhong Agricultural University, 11 (2):* 150~154.

Huang, W. J.; Chen, Y. X., & Wen, Y. X. (1995). Chinese rodents. Shanghai: Fudan University Press.

Huang, Z. G. & Morton, B. (1993). *Mytilopsis sallei* established in Victoria Harbour. *Malacol Rve., 16:* 97~98.

Huang, Z. G. (1984). *Crepidula* *onyx* spreed in Shenzhen Bay. *Marine Science Bulletin, 3 (6):* 92~93.

Huang, Z. G., Morton, B., & Yipp, M. W. (1983). *Crepidula onyx* into and established in Hongkong. *Malacological Review, 16:* 97~98.

Huang, Z. G., Brian M., & Ye, Y. W. (1984). The distribution and the physiological and ecological characteristics of Crepidula onyx in Hongkong. *Acta Oceanologica Sinica, 5 extra edition:* 827~839.

IUCN. (2001). 100 of the World’s Wors84t Invasive Alien Species. Auckland: Invasive Species Specialist Group.

Jin, R. H., Wei, S. Q., & Liang, Y. B. (1991). First exploration to Adaptive area of *Hyphantria cunea* in China. *Plant Quarantine, 5 (4):* 241~246.

Kadoorie Farm & Botanic Garden. (2002). Report of Rapid Biodiversity Assessments at Dinghushan Biosphere Reserve, Western Guangdong, China, 1998 and 2000. South China Forest Biodiversity Report Series: No. 7. KFBG, Hong Kong SAR, ii + 25 pp.

Lever, C. (1997). Naturalized Fishes of the World. San Diego: Academic Press. xxiv + 408.

Li Yanghan. (1998). Flora of China. Beijing: China Agriculture Press.

Li Z & Xie Y. (2002). Invasive Alien Species in China. Beijing: China Forestry Publishing House.211p.

Li, H. M. & Wang, J. F. (1995). Ecology and aquacultural engineering of economic frog. Beijing: China Forestry Publishing House.

Liu, C. X. & Qin, K. J. (1987). Fauna of Liaoning Province · fish. Shenyang: Liaoning Science and Technology Press.

Liu, Z. Y., Jiang, Y., Su, X. Y. Peng, X. F., Wei, H. j., Shi, W. P., & Tang, G. Q. (1998). Biology and control of termites in China. Chengdu: Chengdu University of Science and Technology Press.

Liu, Z., Zhu, S., & Wang, Y. (1995). Successful intorduction of Neosalanx taihuensis Chen into a highland lake, Lake Dianchi, China. In: Timotius, K. H. and Goltenboth, F. (eds.) Tropical Limnology: Tropical Lakes and Reserviors. Proceedings of the Intereational Conference on Tropical Limnology. Indonesia: *Satya Wacana Christian University 2:* 123~128.

Ma, Y. Q. & Lou, W. (1980). Distribution and resource in status of *Ondatra zibethicus* in Xingjiang province. *Journal of Natural Resource, (3):* 78~83.

Ma, Y. Q. (1997). The development of fur breeding in China. Review: A collection of studies on the domestication and breeding of endangered wildlife in China. Harbin: Northeast Forestry University Press.

NATESC. (1998). Handbook of Plant Quarantine. Beijing: China Agriculture Press.

NATESC. (2000). Agriculture Plant Quarantine. Beijing: China Agriculture Press.

Naylor, R. L. (1996). Invasions in agriculture: assessing the cost of the Golden Apple Snail in Asia. *Ambio 25, 7 November:* 443~448.

Northwest Plateau Institute of Biology (NPIB), Chinese Academy of Sciences. (1989). *Qinghai economic fauna.* Xining: Qinghai people’s publishing house.

Pang, X. F. & Tang, C. (1994). The prevention issue of the New invasive pest -- *Oracella acuta*. *Forest Pest and Disease, (2):* 32~34.

Reimer, N. J. (1994). Distribution and impact of alien ants in vulnerable Hawaiian ecosystems. pp. 11~22 in Williams DF (ed.).

Seemann, B. 1857. The Botany of the Voyaye of H. M. S. Herald. London: Reeve & Co.

Tan, W. X. & Zhuo, G. H. (1997). The occurrence regularity and prevention ways of *Liriomyza sativae Blanchard*. *China Vegetables, (1):* 27~28.

Tan, Y. & Tong, H. (1989). The status of the exotic aquatic organisms in China. In: De Silva, S. S. (ed.) Exotic Aquatic Organisms in Asia. Proceedings of the Workshop on Introduction of Exotic Aquatic Organisms in Asia. *Asian Fisheries Society Special Publication 3:* 35~43.

The Animal and Plant Quarantine Bureau (APQB). (1999). Chinese Atlas of The Plant Quarantine Pests. Beijing: China Agriculture Press.

Wang, J. J., Huang, Z. G., Zheng, C. X., & Lin, N. (1999). The population dynamics and structure of invasion species -- *Mytilopsis sallei Reeluz* in Xiamen and Dongshan. *Journal of Oceanography in Taiwan Strait, 18 (4):* 372~377.

Wang, Q. Y. (1992). An Inquiry into Control Tactics of Forest Plant Insects – Three Major Quarantine Objects in China. *Journal of Zhejiang Forestry Science and Technology, 12 (4):* 77~79.

Wang, R. & Ding, J. Q. (1996). The convention of prevent to invasive pests and international biocontrol. View: *Academic meeting of national prevention and control on crops diseases*.

Wang, S. A. (2001). Fauna of Hebei Province · fish. Shijiazhuang: Hebei Science and Technology Press.

Wang, W. P. (1996). The popularization and application of *Hyphantria cunea* control mode. *Forest Pest and Disease, (3):* 44~45.

Wheeler, W. M. (1910). Ants, their Structure, Development and Behavior. New York: Columbia University Press.

Wiktor, A., Chen, D. N., & Wu, M. (2000). Stylommatophoran slugs of China (Gastropoda: Pulmonata) -- Prodromus. *Folia Malacologica. 8(1):* 3~35.

Wu, F. Z. (1982). Species of the genus *Periplaneta burmeister* from China, with reference to their bionomics and economic importance. *Acta Entomologica Sinica, 25 (4):* 416~422.

Wu, J. (2000). The inspection report of Dendroctonus valens LeConte prevention in US, (6).

Wu, S. J. & Gao, L. X. (2002). The bad biodiversity: alien plant species in Hong Kong. *Biodiversity, 10 (1):* 109~118.

Xu H & Qiang S. (2004). Checklist of invasive alien species in China. Beijing: China Environmental Science Press. 432p

Xu, H, G., Sheng, Q., Genovesi, P., Hui, D., Wu, J., Ling, M., ... Guo, J. Y. (2012). An inventory of invasive alien species in China. *Neobiota, 15,* 1-26.

Xu, R. Q., Ding, X. Y., & Zeng, Z. F. (1997). New filed pest -- *Myocastor coypus*. *Plant Protection, 23 (5):* 46~47.

Yang, J. X. (2001). Invasive and Indigenous fishes in Yunnan Province: the study of ways and degree in influencing. View: Wang, S., Xie, B. D., & Xie, Y. Biodiversity Conversion in China (2). Beijing: China Environmental Science Press.

Yang, P. L. (1993). China first coming genus pest in pine -- *Oracella acuta*. *Collecting paper of entomology, 10:* 158.

Yao, G. D., Sun, Z. Z., Guo, L. J., & Lin, H. S. (1995). *Procambarus clarkia* -- occurrence resource in natural waters which suburb of the Shanghai. *Fisheries Science & Technology Information, 22 (2):* 75~77.

Ye, C. Y., Fei, L., & Hu, S. Q. (1993). Rare and economic amphibians in China. Chengdu: Sichuan Science and Technology Press.

Yin, H. F. (2000). The simplification morphological and biological characteristics of *Dendroctonus valens LeConte. Zoological Systematics,25 (1):* 120, 43.

Zhang, G. H., Cao, W. X., & Chen, Y. Y. (1997). Effect of fish stocking on lack ecosystem in China. *Acta Hydrobiologica Sinica, 21 (3):* 271~280.

Zhang, K. M. (1985). Taiwan invasive shellfish -- *Mytilidae*. *Journal of shellfish (Taiwan), (11):* 61~67.

Zhang, L., Yu, H. Y., & Xu, Z. X. (1994). *Lissorbqptrus oryzqphilus Kuschel* and Plant Quarantine (1). *Plant Quarantine, 8(4):* 215~219.

Zhang, R. J. & He, X. F. (1997). Insect Ecogeography and the Control of Dangerous Invasion insect pests. *Ecologic Science, 16 (1):* 83~87.

Zhang, S. F., Liu, Y. P., & Wu, Z. Q. (1998). Beetles Associated With Stored Products in China. Beijing: China Agricultural Science and Technology Press.

Zheng, S. S. (1999). Inspect the biological characteristics of Procambarus clarkia. *Journal of Aquaculture, (4):* 9~10.

Zhou, S. Q. (1995). The synopsis of freshwater fishes of China. Nanjing: Jiangsu Science and technology Press.

Zhou, W. C., Cheng, B. Q., Cai, J. F., & Li, D. P. (1989). Pay strengthen attention to quarantine of the *Achatina fulica Bowditch*. *Plant Quarantine, 3 (1):* 10~13

Zhou, X. H. & Wang, A. M. (1997). Development prospect and ways of Chinese Beaver breeding industry. View: A collection of studies on the domestication and breeding of endangered wildlife in China. Harbin: Northeast Forestry University Press.
